# A novel intelligent bearing fault diagnosis method based on signal process and multi-kernel joint distribution adaptation

**DOI:** 10.1038/s41598-023-31648-y

**Published:** 2023-03-20

**Authors:** Jundi Xiong, Shihai Cui, Haihong Tang

**Affiliations:** 1 Chongqing Electric Power College, Chongqing, 400053 China; 2 Electrified Powertrain Engineering, Changan Ford Automobile Co., Ltd, Chongqing, China; 3 The School of Marine Engineering Equipment, Zhejiang Ocean University, Zhejiang, 316022 China; 4 The Graduate School and Faculty of Bioresources, Mie University, Tus, 514-8507 Japan

**Keywords:** Engineering, Mathematics and computing

## Abstract

The present research on intelligent bearing fault diagnosis assumes that the same feature distribution is used to obtain training and testing data. However, the domain shift (distribution discrepancy) issue generally occurs in both datasets because of different operational conditions. The domain adaptation techniques are preferably applied for fault diagnosis to handle the domain shift issue. Moreover, collecting sufficient testing data or labelled data in real industries is a challenging task. Therefore, the multi-kernel joint distribution adaptation (MKJDA) with dynamic distribution alignment is proposed for bearing fault diagnosis. This method dynamically joins both the marginal and conditional distributions and uses the multi-kernel to solve the non-linear problems to extract the most effective and robust representation for cross-domain issues. Moreover, it runs with the unlabelled task domain to perform the diagnosis by iteratively updating the pseudo code. The experimental results (two public datasets and one experimental dataset) demonstrated that the proposed method (MKJDA) exhibited stable and robust accuracy while conducting bearing fault diagnosis. It can effectively address the most crucial issue: intelligent diagnosis methods must re-train the model when the distribution differs between the source domain (the model is learned) and the target domain (the learned model is applied).

## Introduction

Over the past decade, intelligent fault diagnosis methods^1^, especially data-driven method, were proposed to perform diagnostic decisions intelligently and produce satisfactory diagnosis results without prior expertise^2,3^. However, those methods have several disadvantages before their flexible applications in the industry.

First, most existing intelligent fault diagnosis methods^4,5^ assume that the same distribution is present in the training and testing data. However, the distribution discrepancy^6^ is generally present in both datasets because of the different operating conditions and noise interference. This can lead to the compulsory relearning the diagnostic model with the training data when applied to diverse application conditions, wherein the trained model can guarantee high accuracy. Second, obtaining the labelled data in the real industry is difficult because normal data can be collected in advance^7^. Therefore, an unbalance in the label datasets can also limit the existing data-driven methods^8^, leading to the loss of generalisation ability. Accordingly, it is urgent to devise a new approach to reduce the distribution discrepancy and identify one novel but similar testing task instead of reconstructing and re-training a new diagnosis model from scratch^9,10^.

The transfer learning (TL)^11^, including domain adaptation^12^ and deep transfer learning^13^ can leverage present knowledge from the source domain to achieve target domain tasks^14^. And domain adaptation is the critical technology to minimise the distribution discrepancy between the source and target domain by exploring domain-invariant features to address the domain shift problem. It mainly comprises distribution adaptation^15,16^, feature selection^17^ and subspace learning^18^. Ma^19^ utilised the transfer component analysis (TCA) to perform bearing fault diagnosis and demonstrated that it is a promising strategy for dealing with diverse domain tasks. However, this method ignores the conditional distribution is critical to achieving robust distribution adaptation, even though this distribution achieved better performance in the fault diagnosis process compared with traditional machine learning methods, such as back-propagation neural network (BPNN) and support vector machine (SVM).

For the abovementioned issues, the joint distribution adaptation (JDA)^20^ is an effective and robust strategy for several cross-domain problems, even if the distribution discrepancy is substantially large. First, the JDA applies the nonparametric maximum mean discrepancy (MMD)^21^ to measure the differences in both marginal and conditional distributions. Then, the principal component analysis (PCA) is embedded to construct feature representation. Finally, this method simultaneously reduces the differences in both marginal and conditional distributions of the different domain datasets by leveraging the transferable knowledge from the source domain.

However, some issues exist in bearing fault diagnosis. (i) Raw signals measured from industrial processes exhibit the characteristics of nonlinearity and nonstationary caused by high coupling in the system. (ii) There are mainly three sources of raw signals during machine operation: bursts caused by the defects, inherent vibrations caused by the elastic factors of the bearing, and background noise resulting from different components and other machines. Therefore, the signal generally contains multiple intrinsic oscillatory modes and complex patterns, treated as intensive background noise that causes low energy in the fault features.

The multi-kernel joint distribution adaptation (MKJDA) is a novel diagnosis framework based on JDA to address the need for real industrial applications to improve the accuracy and robustness between different domain datasets. Considering the non-linear and unstable problem in the bearing signal, the multi-kernel function, defined as the convex combination of *d* Gaussian kernels, is introduced into classical MMD to address those challenges. Moreover, the concept of dynamic distribution alignment is introduced into proposed method to quantitatively evaluate the significance of aligning marginal and conditional distributions in DA. In addition, the statistical approach is utilised to enhance the signal–noise ratio (SNR) for the strong background noise. The contributions are organised as follows.

1. A novel proposed method named MKJDA is suggested to simultaneously reduce the differences in both marginal and conditional distributions of the different datasets for bearing fault diagnosis across diverse domains. Moreover, it can also ensure the effective transfer of the trained model from the source domain to a new but similar target domain for achieving highly accurate and robust applications.
2. The MKJDA is applied to three different bearing experimental platforms. It comprises three tasks, namely the different positions of bearing platforms, the diverse fault severity levels and the varying operating conditions, to demonstrate the effectiveness and robustness. Moreover, the experiments indicate that the proposed method exhibits stable and robust accuracy upon application to two public datasets and one industrial dataset compared with the other nine fault diagnosis methods (traditional machine learning, MKJDA with continuous wavelets transform^22^, classical TL, deep learning and deep TL) when there is no label in the target domain in the fault diagnosis process.

## Basic theory

### A basic notation for TL

A basic notation about TL on fault diagnosis is stated in this section. The domain ${\boldsymbol{\mathcal{D}}}$ comprises an *m*-dimensional feature space ${\boldsymbol{\mathcal{X}}}$ and the marginal probability distribution $P\left( {\mathbf{x}} \right)$ (${\mathcal{D}} = \left\{ {{\boldsymbol{\mathcal{X}}},P\left( {\mathbf{x}} \right)} \right\}$, ${\mathbf{x}} \in {\boldsymbol{\mathcal{X}}}$). A task ${\mathcal{T}}$ comprises a *C*_*h*_-cardinality label set ${\mathcal{Y}}$ and a classifier $f\left( {\mathbf{x}} \right)$. ${\mathcal{T}} = \left\{ {{\mathcal{Y}},f\left( {\mathbf{x}} \right)} \right\}$, $\mathcalligra{y} \in {\mathcal{Y}}$ and $f\left( {\mathbf{x}} \right) = Q\left( {{{\mathcalligra{y}|}}{\mathbf{x}}} \right)$ can be interpreted as the conditional probability distribution. Next, a labelled source domain ${\boldsymbol{\mathcal{D}}}^{s} = \left\{ {\left( {{\mathbf{x}}_{i}^{s} ,\mathcalligra{y}_{i}^{s} } \right)} \right\}_{i = 1}^{{n_{s} }}$ and an unlabelled target domain ${\boldsymbol{\mathcal{D}}}^{t} = \left\{ {\left( {{\mathbf{x}}_{i}^{t} } \right)} \right\}_{i = 1}^{{n_{t} }}$ under an assumption that ${\boldsymbol{\mathcal{D}}}^{s}$ and ${\boldsymbol{\mathcal{D}}}^{t}$ are sampled from joint distributions $P\left( {{\boldsymbol{\mathcal{X}}},{\mathcal{Y}}} \right)$ and $Q\left( {{\boldsymbol{\mathcal{X}}},{\mathcal{Y}}} \right)$, respectively (($P_{s} \left( {{\mathbf{x}}^{s} } \right) \ne P_{t} \left( {{\mathbf{x}}^{t} } \right), Q_{s} \left( {\mathcalligra{y}^{s} {|}{\mathbf{x}}^{s} } \right) \ne Q_{t} \left( {\mathcalligra{y}^{t} {|}{\mathbf{x}}^{t} } \right)$). Moreover, the objective is that the joint distribution is applied to match the collective expectations of the features ${\mathbf{x}}$ and labels $\mathcalligra{y}$ through a feature transformation. However, $Q_{t} \left( {\mathcalligra{y}^{t} {|}{\mathbf{x}}^{t} } \right)$ cannot be estimated exactly because there are no labelled data in the target domain. Thus, the pseudo label is proposed to iteratively refine the feature transformation *T*; and classifier ${\text{y}} = f\left( {\mathbf{x}} \right)$ trained on the labelled source data. In other words, a fake target domain dataset ${\mathcal{D}}_{fake}^{t} = \left\{ {\left( {{\mathbf{x}}_{i}^{t} ,{\text{y}}_{i}^{t} } \right)} \right\}_{i = 1}^{{n_{c} }}$ in bearing fault diagnosis based on the assumptions $Q_{t} \left( {\mathcalligra{y}^{t} {|}{\mathbf{x}}^{t} } \right) = Q_{s} \left( {\mathcalligra{y}^{t} {|}{\mathbf{x}}^{t} } \right)$ is constructed to reduce the cross-domain shifts in joint distributions, extract domain-invariant features and minimise target risk with supervision.

### The statistical filter

A challenge in bearing fault diagnosis is presented by the low SNR^23^, which is caused by the strong background noise because of the vibrations of different components and other machines. Therefore, the statistical filter is applied to remove the noise’s negative effects and learn the sensitive information using the TL method. The statistical filter removes noise by calculating mean and standard deviation (as in Eq. (1)) of each part (total *M* parts) and selecting valuable information with the distinction index (*DI*)^24^.

1 $$ DI_{i} = \frac{{\left| {\mu_{1,i} - \mu_{2,i} } \right|}}{{\sqrt {\sigma_{1,i}^{2} + \sigma_{2,i}^{2} } }} \quad i = 1, 2, \ldots , M $$

where $\mu_{1,i}$ and $\mu_{2,i}$ are the mean values of the *i*th spectrum part calculated by the raw signal at normal and abnormal states, respectively. $\sigma_{1,i}$ and $\sigma_{2,i}$ are standard deviations of normal and abnormal states, respectively.

### The Multi-kernel joint distribution adaptation

#### Feature transformation based on principal component analysis

The PCA is applied for dimensionality reduction to learn a transformed feature representation. The input matrix is denoted as ${\boldsymbol{\mathcal{X}}} = \left[ {{\varvec{x}}_{1} , \ldots ,{\varvec{x}}_{n} } \right]$ and the centring matrix is denoted as ${\varvec{H}} = {\varvec{I}} - \frac{1}{n}1$*,* where $n = n_{s} + n_{t}$*.* The objective function of the PCA is to determine an orthogonal transformation matrix **A** to maximise the covariance matrix.

2 $$ \mathop {\max }\limits_{{{\text{A}}^{{\text{T}}} {\text{A}} = {\text{I}}}} {\text{tr}}\left( {{\text{A}}^{{\text{T}}} {\mathcal{X}}{\text{H}}{\mathcal{X}}^{{\text{T}}} {\text{A}}} \right) $$

where $tr\left( \cdot \right)$ denotes the trace of a matrix. This problem can be efficiently solved by eigendecomposition and the *k*-dimensional representation by ${\mathbf{Z}} = {\mathbf{A}}^{{\text{T}}} {\boldsymbol{\mathcal{X}}}$.

#### Joint distribution adaptation

One issue needs attention following feature extraction with PCA: the conditional distributions between the source and target domains should be drawn closer based on reducing the difference in marginal distributions. Furthermore, robust distribution adaptation^25^ must minimise the difference between the conditional distributions $Q_{s} \left( {\mathcalligra{y}^{s} {|}{\varvec{x}}^{s} } \right)$ and $Q_{t} \left( {\mathcalligra{y}^{t} {|}{\varvec{x}}^{t} } \right)$. It is necessary to join distribution adaptation and the marginal probability distribution for the conditional distributions. Unfortunately, the $Q_{t} \left( {\mathcalligra{y}^{t} {|}{\varvec{x}}^{t} } \right)$ cannot directly calculate because there are no labels in the target domain. Even though there are several methods^26^ to match the conditional distributions, they still require a small number of label sets. Thus, the pseudo labels^27^ of the target domain, which can be quickly learned by applying base classifiers^28^ trained on the source domain to unlabelled target domain, are explored to address above issues.

Because the posterior probabilities $Q_{s} \left( {\mathcalligra{y}^{s} {|}{\mathbf{x}}^{s} } \right)$ and $Q_{t} \left( {\mathcalligra{y}^{t} {|}{\mathbf{x}}^{t} } \right)$ are involved to a large extent, the sufficient statistics of the class-conditional distribution $Q_{s} \left( {{\mathbf{x}}^{s} {|}{ }\mathcalligra{y}^{s} } \right)$ and $Q_{t} \left( {{\mathbf{x}}^{t} {|}{ }\mathcalligra{y}^{t} } \right)$ are resorted instead of the posterior probabilities. Therefore, it can essentially match the class-conditional distribution $Q_{s} \left( {{\mathbf{x}}^{s} {|}{ }\mathcalligra{y}^{s} = l} \right)$ and $Q_{t} \left( {{\mathbf{x}}^{t} {|}{ }\mathcalligra{y}^{t} = l} \right)$, where class $l \in \left\{ {1, \ldots ,C_{h} } \right\}$ is present in the label set ${\mathcal{Y}}$. Moreover, the modified MMD is utilised to measure the distance between $Q_{s} \left( {{\mathbf{x}}^{s} {|}{ }\mathcalligra{y}^{s} = l} \right)$ and $Q_{t} \left( {{\mathbf{x}}^{t} {|}{ }\mathcalligra{y}^{t} = l} \right)$, which can address the nontrivial problem of parametrically estimating the probability density for one distribution.

3 $$ \frac{1}{{n_{s}^{\left( l \right)} }}\mathop \sum \limits_{{{\mathbf{x}}_{i} \in {\mathcal{D}}_{\left( l \right)}^{s} }} {\mathbf{A}}^{{\text{T}}} {\mathbf{x}}_{i} - \frac{1}{{n_{t}^{\left( l \right)} }}\mathop \sum \limits_{{{\mathbf{x}}_{j} \in {\mathcal{D}}_{\left( l \right)}^{t} }} {\mathbf{A}}^{{\text{T}}} {\mathbf{x}}_{j}^{2} = {\varvec{B}} $$

4 $$ \left( {M_{l} } \right)_{ij} = \left\{ {\begin{array}{*{20}l} {\frac{1}{{n_{s}^{\left( l \right)} n_{s}^{\left( l \right)} }},} \hfill & {{\mathbf{x}}_{i} ,{\mathbf{x}}_{j} \in {\mathcal{D}}_{\left( l \right)}^{s} } \hfill \\ {\frac{1}{{n_{t}^{\left( l \right)} n_{t}^{\left( l \right)} }},} \hfill & {{\mathbf{x}}_{i} ,{\mathbf{x}}_{j} \in {\mathcal{D}}_{\left( l \right)}^{t} } \hfill \\ {\frac{ - 1}{{n_{s}^{\left( l \right)} n_{t}^{\left( l \right)} }}, } \hfill & {\left\{ {\begin{array}{*{20}c} {{\mathbf{x}}_{i} \in {\mathcal{D}}_{\left( l \right)}^{s} ,{\mathbf{x}}_{j} \in {\mathcal{D}}_{\left( l \right)}^{t} } \\ {{\mathbf{x}}_{j} \in {\mathcal{D}}_{\left( l \right)}^{s} ,{\mathbf{x}}_{i} \in {\mathcal{D}}_{\left( l \right)}^{t} } \\ \end{array} } \right.} \hfill \\ {0,} \hfill & {otherwise} \hfill \\ \end{array} } \right. $$

where ${\mathcal{D}}_{\left( l \right)}^{s} = \left\{ {{\mathbf{x}}_{i} :{\mathbf{x}}_{i} \in {\mathcal{D}}^{s} \wedge \mathcalligra{y}\left( {{\mathbf{x}}_{i} } \right) = l} \right\}$ is the set belonging to class *l* in the source domain;${\varvec{B}} = tr\left( {{\mathbf{A}}^{{\text{T}}} {\boldsymbol{\mathcal{X}}}{\mathbf{M}}_{{\varvec{l}}} {\boldsymbol{\mathcal{X}}}^{{\text{T}}} {\mathbf{A}}} \right)$; $\mathcalligra{y}\left( {{\mathbf{x}}_{i} } \right)$ is the true label of ${\mathbf{x}}_{i}$ and $n_{s}^{\left( l \right)} = \left| {{\mathcal{D}}_{\left( l \right)}^{s} } \right|$. Correspondingly, ${\mathcal{D}}_{\left( l \right)}^{t} = \left\{ {{\mathbf{x}}_{j} :{\mathbf{x}}_{j} \in {\mathcal{D}}^{t} \wedge \widehat{\mathcalligra{y}} \left( {{\mathbf{x}}_{j} } \right) = l} \right\}$ is the set belonging to class *l* in the target domain, $\widehat{\mathcalligra{y}}\left( {{\mathbf{x}}_{j} } \right)$ is the predicted label of ${\mathbf{x}}_{j}$ and $n_{t}^{\left( l \right)} = \left| {{\mathcal{D}}_{\left( l \right)}^{t} } \right|$. Hence, the modified MMD matrix ${\mathbf{M}}_{{\varvec{l}}}$ is expressed as (4).

However, the different significance between marginal and conditional distributions must be evaluated in various cross-domain fault diagnosis tasks. Therefore, the dynamic distribution alignment^29^ is introduced in the proposed method to quantitatively assess the importance of aligning marginal and conditional distributions in DA. Finally, the conditional distributions between two domains are drawn closer under the new feature transformation ${\mathbf{Z}} = {\mathbf{A}}^{{\text{T}}} {\boldsymbol{\mathcal{X}}}$ by minimising Eq. (3) such that Eq. (2) is maximised. This advanced improvement can be robust for DA and sustain changes in conditional distributions. Moreover, it matches the distributions by exploring enough statistics instead of the density estimates^30^. Hence, it can still leverage the pseudo target labels to match the conditional distributions with the modified MMD, as in Eq. (3).

#### Multi-kernel introduced into JDA

Considering the non-linear and unstable problem in the bearing signal^31^, the multi-kernel function (as in Eq. (5)), defined as the convex combination of *d* Gaussian kernels (the RBF kernel), is introduced to evaluate those problems. Theoretically, larger bandwidth values of the RBF kernel can make shrinkage regularisation more critical in the proposed method. When kernel → 0, the optimisation problem is ill-defined. When kernel → ∞, the proposed method cannot construct a robust representation for cross-domain classification. Thus, research classification accuracy is analysed with different values of kernels and indicates that kernel bandwidth ∈ [0.01, 1.0].

5 $$ {\mathbf{K}} \triangleq \left\{ {k = \mathop \sum \limits_{g = 1}^{m} \alpha_{g} k_{g}^{1} :\mathop \sum \limits_{g = 1}^{m} \alpha_{g} = 1,\alpha_{g} \ge 0,\forall g} \right\} $$

where the constraint on the sum of coefficients $\left\{ {\alpha_{g} } \right\}$ is applied to ensure that the derived multi-kernel is a characteristic. Moreover, Eq. (3) is incorporated into Eq. (2) to simultaneously minimise the differences in the marginal and conditional distributions across the source and target domains, as in the following equation:

6 $$ \mathop {\min }\limits_{{{\mathbf{A}}^{{\text{T}}} {\mathbf{KHK}}^{{\mathbf{T}}} {\mathbf{A}} = {\mathbf{I}}}} \mathop \sum \limits_{l = 0}^{{C_{h} }} tr\left( {{\mathbf{A}}^{{\text{T}}} {\mathbf{KM}}_{{\varvec{l}}} {\mathbf{K}}^{{\text{T}}} {\mathbf{A}}} \right) + \lambda {\mathbf{A}}_{F}^{2} $$

7 $$ \begin{gathered} L = {\text{tr}}\left( {{\mathbf{A}}^{{\text{T}}} \left( {{\mathbf{K}}\mathop \sum \limits_{l = 0}^{{C_{h} }} {\mathbf{M}}_{{\varvec{l}}} {\mathbf{K}}^{{\text{T}}} + \lambda {\mathbf{I}}} \right){\mathbf{A}}} \right) \hfill \\ \;\;\;\;\; + {\text{tr}}\left( {\left( {{\mathbf{I}} - {\mathbf{A}}^{{\text{T}}} {\mathbf{K}}{\mathbf{H}}{\mathbf{K}}^{{\text{T}}} {\mathbf{A}}} \right){{\varvec{\Phi}}}} \right) \hfill \\ \end{gathered} $$

where $\lambda$ is the regularisation parameter to guarantee the well-defined optimisation problem. ${{\varvec{\Phi}}} = {\text{diag}}\left\{ {\phi_{n} } \right\}_{n = 1}^{k}$ is denoted as the Lagrange multiplier, and the Lagrange function for Eq. (7).

Setting $\frac{\partial L}{{\partial {\mathbf{A}}}} = 0$ and the generalised eigendecomposition is obtained as in Eq. (8). Therefore, the optimal problem can be solved by finding the optimal adaptation matrix **A**.

8 $$ \left( {{\mathbf{K}}\mathop \sum \limits_{l = 0}^{{C_{h} }} {\mathbf{M}}_{{\varvec{l}}} {\mathbf{K}}^{{\text{T}}} + \lambda {\mathbf{I}}} \right){\mathbf{A}} = {\mathbf{KHK}}^{{\text{T}}} {\mathbf{A\Phi }} $$

## The proposed method

Because the operating conditions in the real industrial processes can cause unobtainable target fault sessions in the fault diagnosis process, leading to a large discrepancy between source and target domains. Therefore, the traditional diagnosis is restricted in real industrial applications because they should re-train the model undergoing different operating conditions. Consequently, one novel proposed transfer diagnosis framework based on KJDA is performed for bearing fault diagnosis on diverse transfer tasks.

### Fault diagnosis procedure

**Figure 1:**
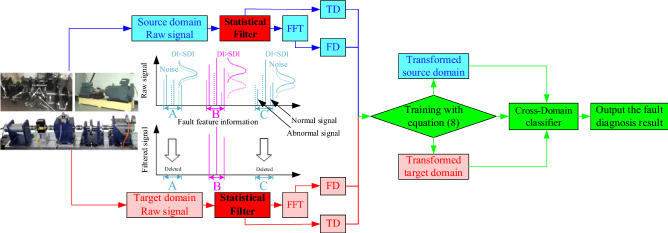
Flowchart of the proposed method (MKJDA).

The flowchart of the novel transfer fault diagnosis method (MKJDA) is shown in Fig. 1. First, the proposed method focuses on the issue under the non-label data. Therefore, enormous amounts of data (with label) are used as the source domain, and the same number of data (non-label) is used to accurately diagnose the target domain. Moreover, the raw signal of source and target domains is treated with the statistical filter, which is also translated into the frequency domain to establish clearer information for fault diagnosis.

Second, the MKJDA approach is presented for effective TL in bearing fault diagnosis facing many conditions and different sessions. This process has four steps based on the treatment of the signal with a statistical filter.

(i) The modified MMD joins the marginal distribution and the conditional distribution according to Eq. (3) and updates the ${\mathbf{M}}_{{\varvec{l}}}$ according to Eq. (4);
(ii) For the non-linear problem in the bearing signal, the multi-kernel (Eq. 5) is utilised to estimate the kernel matrix ${\mathbf{K}}$ in Eq. (6);
(iii) PCA is used to solve generalised eigendecomposition problem in (8) and select the *k* smallest eigenvectors to construct the adaptation matrix **A**;
(iv) Transformed source and target domain data ${\mathbf{Z}} = {\mathbf{A}}^{{\text{T}}} {\mathbf{K}}$ were obtained.

Third, classifier *f* can be learned with the principle of structural risk minimisation (SRM)^32^. Thus, classifier $f$ on $\left\{ {\left( {{\mathbf{A}}^{{\text{T}}} \mathcalligra{k}_{i} ,\mathcalligra{y}_{i} } \right)} \right\}_{i = 1}^{{n_{s} }}$ is trained to update the pseudo target labels $\left\{ {\widehat{\mathcalligra{y}}_{j} : = f\left( {{\mathbf{A}}^{{\text{T}}} \mathcalligra{k}_{j} } \right)} \right\}_{j = 1}^{{n_{t} }}$.

Finally, cross-domain fault diagnosis accuracy is output.

### Comparison methods

This work will compare the proposed TL method with several methods, including traditional neural network, deep learning and TL methods.

1. For the traditional neural network (BPNN and SVM), the input matrix includes the 12 features from the time domain (TD)^33,34^ and 7 features from the corresponding spectrums (FD)^35,36^ in the frequency domain. The raw signal is treated with a statistical filter, and the treated signal is divided into 112 segments equal to the length of 1,024 samples. The TD and TF features calculated from each segment are concatenated into a column in the input matrix. Moreover, there are ten trials for BPNN and SVM because of random initialisation.
2. For fairness, the CWT is used for signal pre-processing, wherein the raw signal is decomposed into several multi-resolution signal levels using a wavelet function (Coiflets) and a threshold selection (minimax). The hard thresholding rule is applied at each level. Finally, the treated signal is put into the improved TL methods (MKJDA) for fault diagnosis.
3. It is necessary to conduct a comparative experiment between MKJDA and deep learning that can discover multiple levels of features from the signal of interest. Thus, existing deep learning methods (stacked auto-encoder [SAE], deep belief nets [DBN] and convolutional neural networks [CNN] in^37^) have been considered plausible methods for remedying the limitations of hand-crafted features and providing greater robustness to intra-class variability caused by noise.
4. In this work, the MKJDA was also compared with traditional TL methods, including TCA and JDA, because MKJDA is inspired by those contrast methods. Moreover, the TCA mainly concentrate on marginal distribution. For fairness of the comparative experiment, 19 statistical features with MKJDA will be extracted from the raw signal employed for the unsupervised DA. Moreover, the deep TL method VGG-16^38^ with statistical filter is applied for comparative experiments to demonstrate the proposed method’s effectiveness.

## Experiments in study 1

To promote the successful applications of the proposed TL method, the open-access datasets, including with four different health conditions (i.e., normal [N], defect in the outer race [O], defects in the inner race [I] and defects in the roller [R]), were acquired from the tested motor bearings (6205-2RS JEM SKF) in Case Western Reserve University^39^. Moreover, the electro-discharge machining is applied to set a single point failure with three severity levels of faults (the fault diameters of 7 mils, 14 mils and 21 mils). Three accelerometers were placed respectively in the drive end (DE) and fan end (FE); the sampling frequency was 12 kHz. Moreover, the test bearing supports the motor shaft and motor loads of 0–2 horsepower (hp).

### The metrics for fault diagnosis

In this paper, four metrics were evaluated by considering each category for performance evaluation, as defined in (9)–(12).

9 $$ specificity = {{TN} \mathord{\left/ {\vphantom {{TN} N}} \right. \kern-0pt} N} $$

10 $$ precision = {{TP} \mathord{\left/ {\vphantom {{TP} {\left( {TP + FP} \right)}}} \right. \kern-0pt} {\left( {TP + FP} \right)}} $$

11 $$ recall = {{TP} \mathord{\left/ {\vphantom {{TP} P}} \right. \kern-0pt} P} $$

12 $$ F = \frac{2TP}{{2TP + FP + FN}} $$

where *P* = *TP* + *FN*; *N* = *FP* + *TN*; true positive (*TP*) represents correctly classified positive samples, false positive (*FP*) is misclassified positive samples, true negative (*TN*) is correctly classified negative samples and false negative (*FN*) is misclassified negative samples.

### Raw signal pre-processing with statistical filter

**Figure 2:**
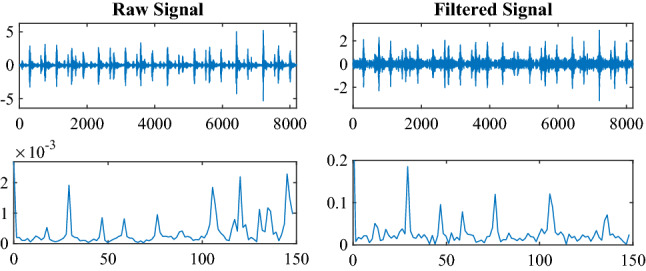
Raw signal and filtered signals.

In this section, the objective is to prove the effectiveness of the statistical filter for treating noise by comparing the spectrum of raw and filtered signals. Moreover, the signals of the outer (1 hp) are treated as an example because of the length of the article. Figure 2 shows the spectrum’s energy in the filtered signal is stronger than in the raw signal. Moreover, the fault feature frequency of the filtered signal is clearer than that of the raw signal. Therefore, the statistical filter can remove background noise and enhance the SNR for fault diagnosis based on TL.

### Experiment results

The positions of accelerometers, severity level and motor load can all affect the characteristics of specific faults. This phenomenon causes a domain discrepancy in the real industry fault diagnosis when the target domain is not well represented in the source domain, wherein sufficient data are used to train the diagnosis model. Therefore, the three transfer tasks are as follows.

#### Task I

There are two kinds of datasets (DE and FE) under different accelerometers installed in different experimental positions (variable positions) to simulate the TL task. For clarity, D → F is defined as the transfer task from the source dataset D to the target dataset F. The source data D (1797 rpm) has four different kinds of health states, which are collected from the accelerometers installed in the DEIn contrast. The target dataset F comprised the same signal collected from the accelerometers installed in the FE. Table 1 enlists detailed information on four health states.

**Table 1:** Detailed information of the dataset for task I (0 hp).

Task	State	Label	Label source	Unlabelled target
–	Normal (N)	0	500	500
0.021	Outer (O21)	1	500	500
0.021	Inner (I21)	2	500	500
0.021	Roller (R21)	3	500	500

**Figure 3:**
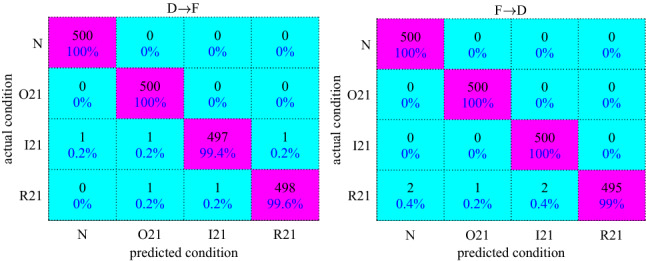
The experiment results of Task I (D → F and F → D).

Figure 3 and Table 2 present the experimental findings of the proposed method (MKJDA) for better interpretation. Both vividly observe that the proposed method achieves much better performance through the hot map and statistical significance of four metrics. For Task I, the average classification accuracy of MKJDA is over 99%. The maximum and minimum accuracies are 100% and 99.4%, respectively. Therefore, it verifies that the MKJDA can construct a more effective and robust representation for cross-domain classification tasks of the bearing fault diagnosis because the dynamic distribution alignment and multi-kernel MMD are introduced into the TL method. Similarly, the statistical filter also enhances the SNR.

**Table 2:** The list of four metrics for MKJDA.

	*Specificity* (%)	*Precision* (%)	*Recall* (%)	*F* (%)
D → F	98.96	99.01	99.34	99.23
F → D	99.03	99.34	99.23	99

Moreover, comparative experiments (shown in Table 3) demonstrated that MKJDA outperforms the other methods by comparing the metric (*F*%) because it successfully matches the conditional distributions and marginal distributions by exploring only sufficient statistics when applied for bearing fault diagnosis.

(i) The traditional machine learning (BPNN and SVM) is limited with data distribution;
(ii) Signal process (CWT) is restricted to the nonstationary signal because it has a fixed time–frequency window, wherein it cannot conduct adaptive analysis with abundant frequency information;
(iii) Classical transfer (TCA and JDA) has no ability for dynamic distribution alignment and multi-kernel MMD. Moreover, the proposed method’s accuracy is comparable to that of the deep transfer method (VGG-16), and its operating efficiency is better than that of VGG-16, DMAEAM-DDA^40^ and JSWD^41^.
(iv) Deep learning has better accuracy than that of classical transfer. However, it is worse than that of proposed method because dynamic distribution alignment and multi-kernel MMD are introduced into the transfer learning method.

**Table 3:** Comparative results between different methods (*F*%).

	MKJDA	Traditional machine learning		Signal process	traditional transfer		Deep learning			Novel transfer		
		BPNN	SVM	MKJDA + CWT	TCA	JDA	SAE	DBN	CNN	VGG-16	DMAEAM-DDA	JSWD
D → F	99.01	37.56	60.39	80.78	60.12	65.23	74.25	70.56	83.45	99.34	99.56	99.02
F → D	99	38.34	58.48	83.65	59.49	66.08	72.12	73.85	82.56	99.46	99.61	99.15

#### Task II

The datasets (task II) are selected from three fault diameters in DE for demonstration. The datasets with 0 hp are named ‘1’–‘6’. Table 4 presents the details of Task II across different severity levels with 0 hp.

**Table 4:** Designed transfer tasks II across diverse severity levels (0 hp).

Transfer tasks	Source domain	Target domain	Label source	Unlabelled Target	Conditions
1 → 2	7 miles	14 miles	500	500	Labels (0–3)
2 → 1	14 miles	7 miles	500	500	
3 → 4	14 miles	21miles	500	500	
4 → 3	21 miles	14 miles	500	500	
5 → 6	7 miles	21 miles	500	500	
6 → 5	21 miles	7 miles	500	500	

**Figure 4:**
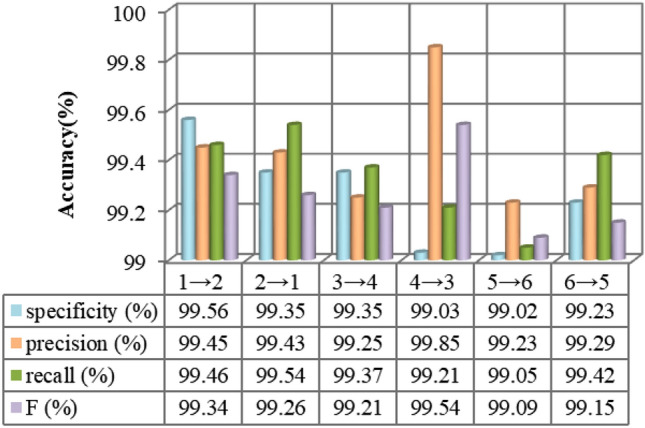
The experiment results of MKJDA (Task II).

Figure 4 shows that the MKJDA has better average accuracy (over 99.35%) and the highest accuracy (99.85%) while performing different transfer tasks in the comparison of four metrics, namely *specificity*, *precision*, *recall* and *F*. This occurs because the proposed method simultaneously reduces the differences in both marginal and conditional distributions of the different datasets for bearing fault diagnosis across diverse domains.

**Figure 5:**
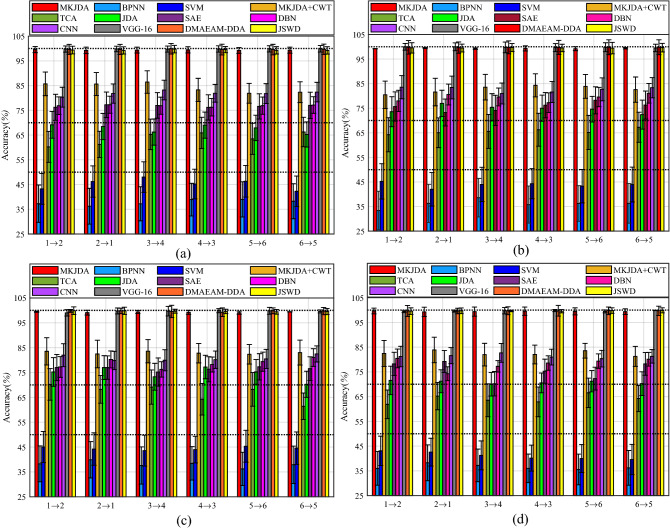
The comparative experimental results (task II). (**a**) *Specificity* (%); (**b**) *precision* (%); (**c**) *recall* (%); and (**d**) *F* (%).

Furthermore, the comparative experiments (shown in Fig. 5) demonstrated that the MKJDA outperforms the other methods in the comparison of the four metrics. The traditional machine learning methods (BPNN and SVM) have the worst accuracy (lower than 50%) compared with other methods, even though the signal is treated with a statistical filter. Furthermore, the CWT is applied for signal treatment, and the accuracy is worse than the proposed method because of the drawbacks of the CWT. the TL methods, including TCA and JDA, also do not have ideal accuracy (lower than 80%) while performing the bearing fault diagnosis with diverse severity levels. In addition, the deep learning method (SAE, DBN and CNN) has no better performance facing the different source domains even though it has strong data mining capabilities. The deep TL method (VGG-16) was also applied for a comparative experiment to demonstrate the proposed method’s effectiveness that has high accuracy.

#### Task III

Task III aims to investigate the proposed method’s ability to diagnose bearing faults across diverse operating conditions. For a bearing fault, four fault state data (21 mils and DE) are collected from motor loads 0, 1, 2 and 3, which are regarded as datasets 0, 1, 2 and 3, respectively. For instance, in 1, the source dataset 0 contains the faults (N, O, I and R) under load 0. In contrast, the four faults under load 1 are from target dataset 1. Table 5 presents the details of the designed transfer task III across diverse operating conditions for bearing fault diagnosis.

**Table 5:** Designed transfer tasks across different motor loads.

Tasks III	Source domain	Target domain	Label source	Unlabelled target	Conditions
1	Load 0	Load 1	500	500	Labels (0–3)
2	Load 1	Load 0	500	500	
3	Load 0	Load 2	500	500	
4	Load 2	Load 0	500	500	
5	Load 0	Load 3	500	500	
6	Load 3	Load 0	500	500	
7	Load 1	Load 2	500	500	
8	Load 2	Load 1	500	500	
9	Load 1	Load 3	500	500	
10	Load 3	Load 1	500	500	
11	Load 2	Load 3	500	500	
12	Load 3	Load 2	500	500	

**Figure 6:**
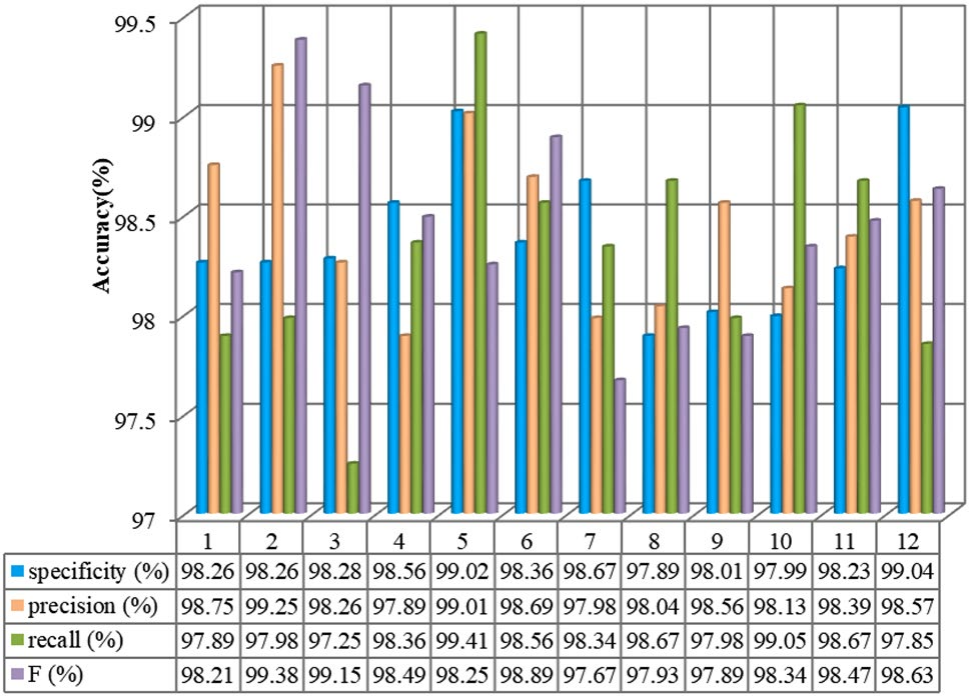
The experiment results of MKJDA (Task III).

Figure 6 shows that the MKJDA has better average accuracy (over 97.95%) and the highest accuracy (99.41%) while facing the different transfer tasks in the comparison of four metrics, namely *specificity*, *precision*, *recall* and *F*. Result is achieved because the proposed method simultaneously reduces the differences in both marginal and conditional distributions of the different datasets for bearing fault diagnosis across diverse domains.

**Figure 7:**
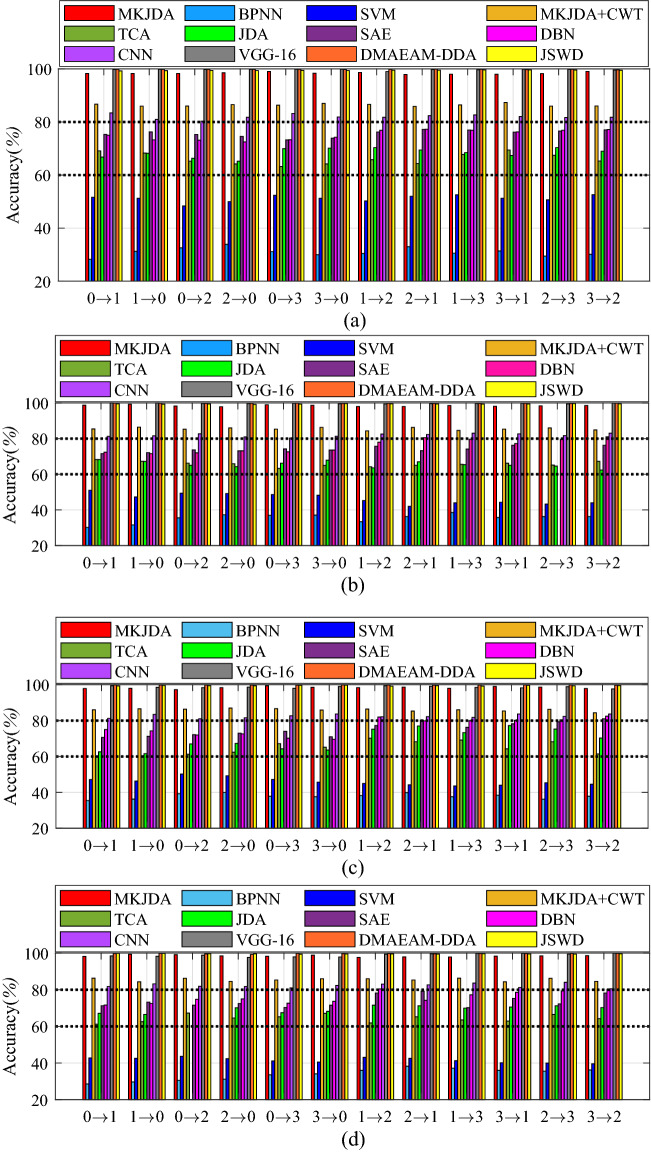
The comparative experimental results (task III). (**a**) *Specificity* (%); (**b**) *precision* (%); (**c**) *recall* (%) and (**d**) *F* (%).

Furthermore, the comparative experiments (Fig. 7) demonstrated that the MKJDA outperforms other methods in the comparison of the four metrics. The traditional machine learning methods (BPNN and SVM) have the worst accuracy (lower than 50%) compared with other methods, even though the signal is treated with a statistical filter. Furthermore, the CWT is applied for the signal treatment, and the accuracy (lower than 85%) is worse than the proposed method because of the drawbacks of the CWT. The TL methods, including TCA and JDA, also do not have ideal accuracy (lower than 80%) while performing the bearing fault diagnosis with diverse severity levels. In addition, the deep learning method did not perform better under different source domains, even with superior data mining capabilities. The three TL methods (VGG-16 DMAEAM-DDA and JSWD) are also applied for comparative experiments to demonstrate the proposed method’s effectiveness. The reason is that the different loads greatly influence the bearing signal feature, directly leading to differences in both marginal and conditional distributions of the source and target domains.

#### Discussion

The reasons for the above experimental results can be summarised as follows:

1. The traditional machine learning method (BPNN and SVM) has the worst accuracy because they ignored the distribution differences and treated the source and target domains as domains subjected to the same distribution. Although classical TL methods (JDA and TCA) and deep learning methods (SAE and DBN) achieve better results compared to BPNN and SVM, the results of those methods are not ideal (< 75%) because they also fail to narrow the distribution difference between the source and target domains when applied to different bearing fault diagnosis. The proposed method with CWT also has unsuitable accuracy because the main disadvantage of CWT depends on professional knowledge and experiments to realise hand-crafted features for classification. Moreover, the proposed method has the same accuracy as three TL, but the efficiency will be better because it is a shallow network.
2. The proposed method combines classical JDA and the novel distance metric MMD, wherein the multi-kernel is introduced into the classical MMD to overcome non-linear and unstable problems in the bearing signal for reducing differences in both marginal and conditional distributions of the different datasets for bearing fault diagnosis across diverse domains. Moreover, novel method also ensures that the trained model from the source domain can be transferred effectively to a new but similar target domain to achieve high accuracy and robust applications under different noises.

### Analysis of robustness

#### Influence of model parameters

The parameters are determined by multiple experiments and taking the best experimental results. Due to the space limitation of the article, we take Task I ~ Task III as an example to explain. If it is necessary to add it into the manuscript, please give us a chance to add relevant content to the original manuscript. And the detailed information is discussed as follows.

**Figure 8:**
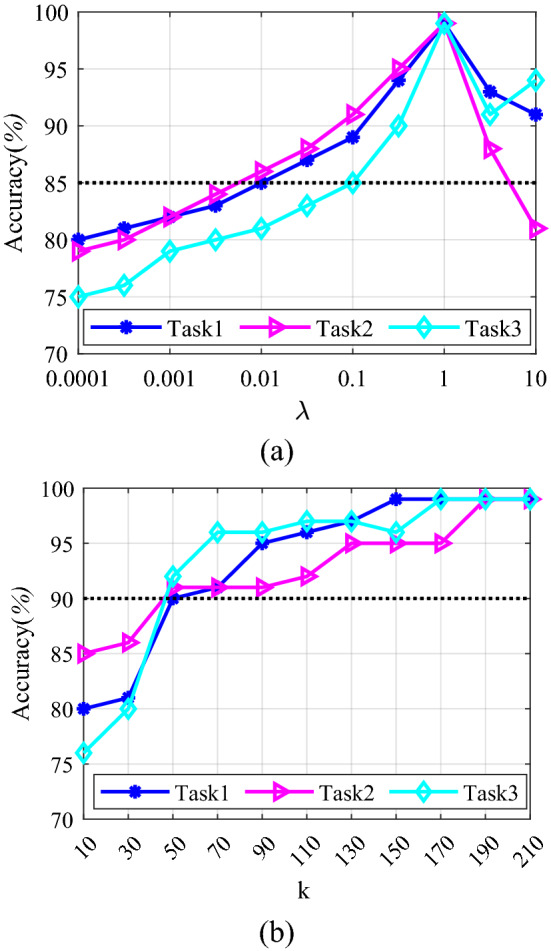
The parameter sensitivity of MKJDA: (**a**) regularization parameter λ; (**b**) subspace bases k.

As shown in Fig. 8b, MKJDA with varying values of k. It can be chosen such that the low-dimensional representation is accurate for data reconstruction. Thus choose k ∈ [50, 210]. Moreover, as shown in Fig. 8a, λ ∈ [0.001, 1.0] can be optimal parameter values, where MKJDA generally does much better than the baselines.

#### Influence of noise

It is crucial to evaluate the robustness of MKJDA against noise to facilitate real-world applications due to noise effects. Additive Gaussian white noise (13) was injected into the raw signals to construct new signals with different SNRs^42^.

13 $$ SNR\;({\text{dB}}) = 10\lg \left( {{{P_{signal} } \mathord{\left/ {\vphantom {{P_{signal} } {P_{noise} }}} \right. \kern-0pt} {P_{noise} }}} \right) $$

**Figure 9:**
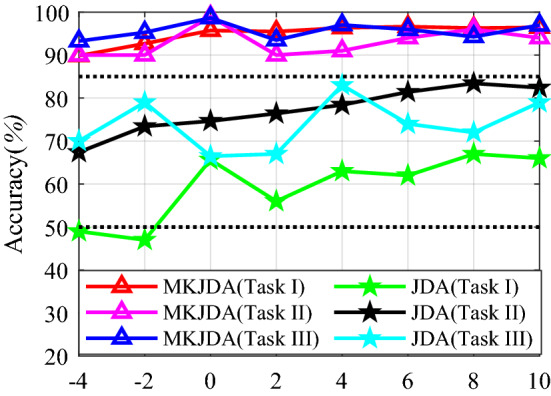
Experimental results between MKJDA and JDA based on signals with the different SNRs.

In this section, the SNRs range from − 4 to 10 dB and the evaluation results are shown in Fig. 9. It is apparent that MKJDA significantly outperforms JDA (< 85%), which has better average testing performance (> 89%) within all considered SNR levels in triple tasks. Specifically, it has stable precision and increased performance from 90 to 98.95%, whereas JDA has evident fluctuations. Moreover, the maximum difference reaches 32% at an SNR of − 2 dB; furthermore, the difference reaches 20% at an SNR of 4 dB as the signal’s power is stronger than that of the noise.

## Experiments in study 2 and study 3

### Transfer task in study 2

**Figure 10:**
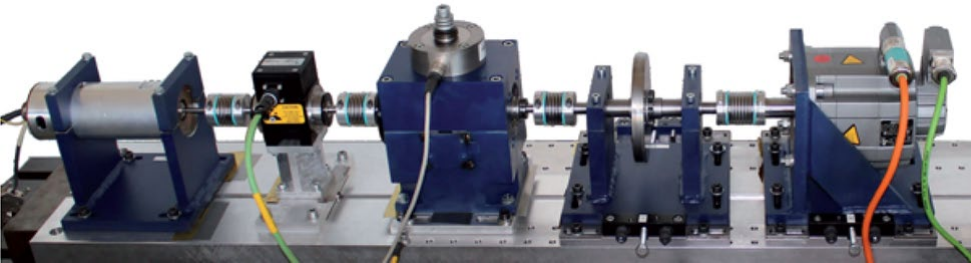
Mechanical setup of the test rig.

In this section, the proposed MKJDA is applied to the bearing fault diagnosis provided by the KAt DataCenter at Paderborn University^39^. The test rig (Fig. 10) is a modular system generating the measurement data required to analyse corresponding signature and damage characteristics derived from motor current signals. The essential components of the test rig are the drive motor (a permanent magnet synchronous motor) acting as a sensor, a torque measurement shaft, the test modules and a load motor (synchronous servo motor).

This dataset contains signals including the normal signal (N), outer fault (O) and inner fault (I). These faults include artificially damaged bearings (electric discharge machine, drilling and manual electric engraving) and realistically damaged bearings (pitting or plastic deformation). The bearings run with three loads, and the sampling frequency and time are 64 kHz and 4 s, respectively. Table 6 presents detailed information on the three tasks (A, B and C). The data treatment is presented in sections "Comparison Methods" and "Raw Signal Pre-processing with Statistical Filter".

**Table 6:** The three tasks in KAt bearing dataset (1500 rpm).

State	Load (Nm)	Radial force	Task
N, O and I	0.7	400	A
	0.1	1000	B
	0.7	1000	C

Table 7 shows that the proposed method has more stable accuracy (maximum difference accuracy is 0.7%) than the other three methods (BPNN: 5%, SVM: 3%, TCA: 11%, JDA: 5%, SAE: 7%, SAE: 9%, CNN: 3% and CWT: 3%). Moreover, it has the best average accuracy (99.5%) among nine methods (62.8%, 67.5%, 83.7%, 82.3%, 99.97%, 90.2%, 90%, 92% and 87%). Thus, it proves that the MKJDA has better generalisation and robustness to address the different fault diagnosis tasks in various domains. Moreover, this phenomenon occurs because the MKJDA joins both the marginal and conditional distributions and uses the multi-kernel to solve the non-linear problem to extract the most effective and robust representation for cross-domain problems. It can also reduce the discrepancy in conditional distributions in each iteration to enhance the classification performance by iteratively refining the pseudo labels. Therefore, the MKJDA is an effective TL method for bearing fault diagnosis with stable accuracy.

**Table 7:** The experimental results with three tasks.

Methods	A → B	B → A	A → C	C → A	B → C	C → B	Average
BPNN	61%	63%	60%	65%	62%	63%	62.8%
SVM	65%	66%	68%	65%	67%	68%	67.5%
TCA	80%	85%	86%	91%	80%	80%	83.7%
JDA	81%	83%	83%	85%	82%	80%	82.3%
SAE	85%	90%	92%	90%	92%	92%	90.2%
DBN	93%	92%	84%	86%	93%	92%	90%
CNN	91%	93%	92%	90%	93%	92%	92%
CWT	88%	89%	86%	87%	88%	89%	87%
VGG-16	99.8%	99.7%	99.7%	99.5%	99.8%	99.7%	99.97%
DMAEAM-DDA	99.86%	99.84%	99.71%	99.79%	99.89%	99.72%	99.82%
JSWD	99.46%	99.78%	99.85%	99.68%	99.65%	99.68%	99.73%
**MKJDA**	**99.1%**	**99.5%**	**99.6%**	**99.8%**	**99.5%**	**99.5%**	**99.5%**

### Transfer task in study 3

#### Experimental results

**Figure 11:**
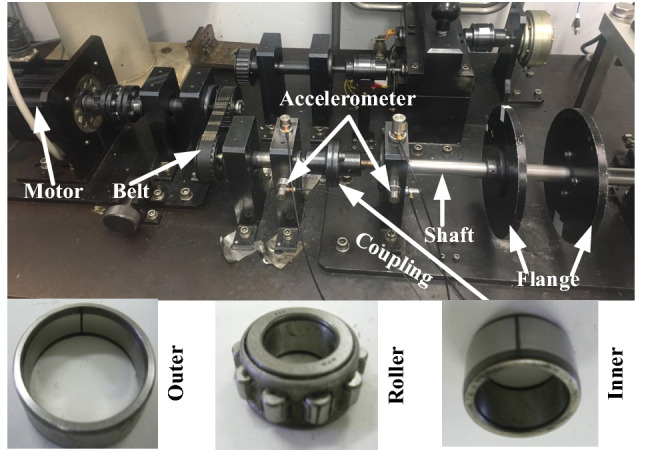
Mechanical setup of industrial process.

There are five accelerometers (PCB MA352A60) must be mounted in the three directions (horizontal, vertical and axial directions) of two bearing housing to acquire the signals with a sampling frequency of 10 kHz and a sampling time of 10 s (shown in Fig. 11). Moreover, the signals measured with the accelerometer are transformed into an oscilloscope (Scope Coder DL750) after being magnified by a sensor signal conditioner (PCB ICP Model480C02). There are four kinds of states in the described experimental machine, namely the normal (N), the single defect on the outer (O) with 0.7 mm × 0.25 mm (width × depth), the single defect on the inner (I) with 0.7 mm × 0.15 mm (width × depth), and the single defect on the roller (R) with 0.7 mm × 0.15 mm (width × depth). Moreover, the rotational speed is set with values of 1200 rpm, 900 rpm and 600 rpm.

Table 8 presents the details of the task design. The data are treated as in Sections "Comparison Methods" and "Raw Signal Pre-processing with Statistical Filter" for fairness. Table 9 shows that the proposed method has more stable accuracy (maximum difference accuracy is 1.5%) than the other three methods (BPNN: 4%, SVM: 8%, TCA: 5%, JDA: 6%, VGG-16: 0.5%, DMAEAM-DDA: 0.35%; JSWD: 0.16; SAE: 4%, DBN: 2%, CNN: 4% and CWT: 4%). Moreover, it has the best average accuracy (99%) among all methods (51.5%, 62%, 81%, 83.6%, 88.6%, 90%, 91%, 85%, 99.95%, 99.95%, and 99.76%). Hence, this outcome proves that the MKJDA has better generalisation and robustness to address the different fault diagnosis tasks in various domains when applied in the relevant industry. Furthermore, it occurs because the MKJDA joins both the marginal and conditional distributions and utilises the multi-kernel to solve the non-linear problem and extract the most effective and robust representation for cross-domain problems. It can also reduce the discrepancy in conditional distributions in each iteration to improve the classification performance by iteratively refining the pseudo labels. Moreover, the dynamic distribution alignment and multi-kernel MMD are introduced into the TL method. The statistical filter also improves the SNR. Therefore, the MKJDA is an effective TL method for bearing fault diagnosis with stable and high accuracy compared with nine advanced methods.

**Table 8:** Designed transfer tasks for the experiments.

Transfer tasks	Source domain	Target domain	Label source	Unlabelled target	Conditions
1 → 2	1200 rpm	900 rpm	500	500	Labels (0–3)
2 → 1	900 rpm	1200 rpm	500	500	
3 → 4	900 rpm	600 rpm	500	500	
4 → 3	600 rpm	900 rpm	500	500	
5 → 6	1200 rpm	600 rpm	500	500	
6 → 5	600 rpm	1200 rpm	500	500

**Table 9:** The experimental results with three tasks.

Methods	1 → 2	2 → 1	3 → 4	4 → 3	5 → 6	6 → 5	Average
BPNN	49% ± 7.56	50% ± 7.23	48% ± 6.89	51% ± 6.59	47% ± 7.11	51% ± 7.09	51.5% ± 7.12
SVM	59% ± 6.23	61% ± 7.01	63% ± 6.33	67% ± 6.18	62% ± 5.99	64% ± 6.18	62% ± 6.52
TCA	79% ± 4.23	78% ± 4.78	82% ± 4.65	78% ± 4.55	83% ± 5.12	80% ± 4.89	81% ± 5.26
JDA	81% ± 5.26	79% ± 5.16	84% ± 5.18	85% ± 5.10	80% ± 5.09	84% ± 5.21	83.6% ± 5.16
SAE	86% ± 3.56	88% ± 3.46	90% ± 3.45	89% ± 3.52	87% ± 3.49	86% ± 3.47	88.6% ± 3.48
DBN	89% ± 4.01	91% ± 3.89	89% ± 3.98	90% ± 3.59	89% ± 4.02	89% ± 3.95	90% ± 3.89
CNN	91% ± 3.01	90% ± 3.21	93% ± 3.11	93% ± 2.95	90% ± 2.96	89% ± 3.01	91% ± 2.89
CWT	83% ± 4.25	84% ± 4.21	83% ± 4.11	87% ± 4.89	85% ± 5.01	86% ± 4.76	85% ± 4.83
VGG-16	99.8% ± 1.23	99.5% ± 2.01	99% ± 1.56	100% ± 1.01	99.8% ± 1.11	99.5% ± 1.23	99.95% ± 1.63
DMAEAM-DDA	99.78 ± 1.56	99.81 ± 1.23	99.85 ± 0.56	99.86 ± 0.89	99.76 ± 0.56	99.83 ± 1.32	99.95 ± 1.11
JSWD	99.65 ± 2.01	99.62 ± 2.06	99.89 ± 2.12	99.56 ± 2.17	99.68 ± 1.36	99.86 ± 2.01	99.76 ± 1.96
**MKJDA**	**99%** ± 1.24	**98%** ± 1.31	**99.5%** ± 1.19	**99.4%** ± 1.25	**99%** ± 1.18	**99%** ± 1.23	**99%** ± 1.06

#### Comparisons of uncertainty distribution

In^43^, an out-of-distribution (OOD) is a challenging issue that may induce the model to produce unreliable and unsafe decision for unforeseen machine data since unseen machine faults are often from unknown distributions in the real applications. Therefore, there are two unseen faults to be considered in this case^44^. One is about the loose fault in the bearing chock (CL). The other is related to the shaft, namely, shaft misalignment (M), caused by the shaft that deviates at an angle from the centre line. There are three diagnostic experiments are designed, whose details are described in Table 10. All the unseen faults are regarded as one label. Moreover, there are two novel metrics, including the false alarm rate (FAR) and the missing alarm rate (MAR)^41^, to value the performance of four methods (VGG-16, DMAEAM-DDA, JSWD and MKJDA).

**Table 10:** Descriptions of industrial experiments.

	Seen conditions	Unseen faults	Training samples	Testing samples
No.1	N, O, I and R Labels 0,1,2,3	CL Label 4	4 × 800	5 × 500
No.2	N, O, I and R Labels 0,1,2,3	M Label 4	4 × 800	5 × 500
No.3	N, O, I and R Labels 0,1,2,3	CL and M Label 4	4 × 800	5 × 500

The purpose of this experiment is to demonstrate the effectiveness of the proposed method which identify unseen faults and give trustworthy diagnostic decisions with respect to the other three methods. And the experimental results are shown in Table 11. And it is obvious that the proposed method has the best diagnostic performances compared with the other three methods. Specially, the MARs is an important metric to evaluate trustworthiness of diagnosis results, which can accurately identify the unseen faults and even unknown OOD to make warning for potential expert intervention. Therefore, it should be maintained as low as possible.

**Table 11:** Diagnostic results of experiments 1–3 considering trustworthy analysis.

	Experiment 1			Experiment 2			Experiment 3		
	Accuracy	FAR	MAR	Accuracy	FAR	MAR	Accuracy	FAR	MAR
VGG-16,	86.56	10.6	15.23	88.25	8.9	20.49	79.46	10.86	38.65
DMAEAM-DDA,	85.59	11.23	21.36	87.12	11.86	14.37	76.45	12.56	29.94
JSWD	87.25	10.25	26.56	83.89	8.50	25.36	80.25	15.43	24.89
**MKJDA**	**90.56**	**8.4**	**0**	**91.25**	**8.6**	**0**	**91.86**	**8.1**	**0**

## Conclusion

The proposed method (MKJDA) is applied for machinery fault diagnosis to address the domain shift problem, which provides reliable and stable diagnosis performance. The effectiveness and superiority of MKJDA are demonstrated by comparisons with several methods in three datasets including two public dataset and one experimental dataset. The results demonstrated that MKJDA is an effective and robust method for various bearing fault cross-domain issues because it simultaneously adapts marginal and conditional distributions during the diagnosis process. In addition, this merit warrants that the learned diagnosis model from the source domain can be transferred effectively to new but similar applications, effectively. It resolves an issue that intelligent diagnosis model should be re-trained when the distribution differs between the source domain (model is learned) and the target domain (model is applied). In the future, the research content will focus on deep transfer learning and its improvement to perform low-speed machinery fault diagnosis.

## References

[1] Neupane D, Seok J (2020). Bearing fault detection and diagnosis using case western reserve university dataset with deep learning approaches: a review. IEEE Access.

[2] Zhang W, Li C, Peng G (2018). A deep convolutional neural network with new training methods for bearing fault diagnosis under noisy environment and different working load. Mech. Syst. Signal Process..

[3] Tang H, Liao Z (2019). Intelligent fault diagnosis for low-speed roller bearings based on stacked auto-encoder. Int. J. Cond. Monit. Diagn. Eng. Manag..

[4] Eren L, Ince T (2019). A generic intelligent bearing fault diagnosis system using compact adaptive 1D CNN classifier. J. Signal Process. Syst..

[5] An Z, Li S (2020). A novel bearing intelligent fault diagnosis framework under time-varying working conditions using recurrent neural network. ISA Trans..

[6] Tang H, Liao Z (2020). Stepwise intelligent diagnosis method for rotor systemwith sliding bearing based on statistical filter and stacked auto-encoder. Appl. Sci..

[7] Zhang Z, Chen H (2020). Unsupervised domain adaptation via enhanced transfer joint matching for bearing fault diagnosis. Measurement.

[8] Pan T, Chen J (2020). Intelligent fault identification for industrial automation system via multi-scale convolutional generative adversarial network with partially labeled samples. ISA Trans..

[9] Li X, Zhang W (2019). Cross-domain fault diagnosis of rolling element bearings using deep generative neural networks. IEEE Trans. Ind. Electron..

[10] Lei Y, Yang B, Jiang X, Jia F, Li N, Nandi AK (2020). Applications of machine learning to machine fault diagnosis: A review and roadmap. Mech. Syst. Signal Process..

[11] Pan S, Yang Q (2009). A survey on transfer learning. IEEE Trans. Knowl. Data Eng..

[12] Xiao Y, Shao H, Han S, Huo Z, Wan J (2022). Novel joint transfer network for unsupervised bearing fault diagnosis from simulation domain to experimental domain. IEEE/ASME Trans. Mechatron..

[13] Han T, Liu C, Yang W, Jiang D (2018). Deep transfer network with joint distribution adaptation: A new intelligent fault diagnosis framework for industry application. ISA Trans..

[14] Yang B, Lei Y (2019). An intelligent fault diagnosis approach based on transfer learning from laboratory bearings to locomotive bearings. Mech. Syst. Signal Process..

[15] Duan L, Tsang I (2012). Domain transfer multiple kernel learning. IEEE Trans. Pattern Anal. Mach. Intell..

[16] Cao H, Shao H, Zhong X, Deng Q, Yang X, Xuan J (2022). Unsupervised domain-share CNN for machine fault transfer diagnosis from steady speeds to time-varying speeds. J. Manuf. Syst..

[17] Li, J., Zhao, J. & Lu, K. Joint feature selection and structure preservation for domain adaptation, in *IjCAI* 1697–1703 (2016).

[18] Sun, B. and Saenko, K. Deep coral: Correlation alignment for deep domain adaptation, in *European Conference on Computer Vision* 443–450 (Springer, 2016).

[19] Ma P, Zhang H (2020). A diagnosis framework based on domain adaptation for bearing fault diagnosis across diverse domains. ISA Trans..

[20] Long, M., Wang, J., et al. Transfer feature learning with joint distribution adaptation, in *Proceedings of the IEEE International Conference on Computer Vision* 2200–2207 (2013).

[21] Tang, H., Liao, Z., et al. A novel convolutional neural network for low-speed structural fault diagnosis under different operating condition and its understanding via visualisation. *IEEE Trans. Instrum. Meas.* **70** (Art no. 3501611), 1–11 (2021).

[22] Cheng Y, Lin M, Wu J, Zhu H, Shao X (2021). Intelligent fault diagnosis of rotating machinery based on continuous wavelet transform-local binary convolutional neural network. Knowl. Based Syst..

[23] Liu H, Wang Z (2021). Improving the signal-to-noise-ratio of free induction decay signals using a new multilinear singular value decomposition-based filter. IEEE Trans. Instrum. Meas..

[24] Tang H, Liao Z (2021). Stepwise intelligent diagnosis method for rotor system with sliding bearing based on statistical filter and stacked auto-encoder. Appl. Sci..

[25] Wang Q, Michau G (2021). Missing-class-robust domain adaptation by unilateral alignment. IEEE Trans. Ind. Electron..

[26] Sanodiya R, Mathew J (2019). A framework for semi-supervised metric transfer learning on manifolds. Knowl. Based Syst..

[27] Song Y, Li Y (2020). Re-training strategy-based domain adaption network for intelligent fault diagnosis. IEEE Trans. Ind. Inf..

[28] Liao Y, Huang R (2020). Deep semi-supervised domain generalisation network for rotary machinery fault diagnosis under variable speed. IEEE Trans. Instrum. Meas..

[29] Wang, J., Feng, W., Chen, Y., Yu, H., Huang, M., and Yu, P. S. Visual domain adaptation with manifold embedded distribution alignment, *in Proceedings of the 26th ACM International Conference on Multimedia* 402–410, October (2018).

[30] Zhao M, Tian Z (2019). Fault diagnosis on wireless sensor network using the neighborhood kernel density estimation. Neural Comput. Appl..

[31] Bao B, Liu C (2012). Inductive robust principal component analysis. IEEE Trans. Image Process..

[32] Vladimir NV, Vlamimir V (1998). Statistical Learning Theory. Wiley.

[33] Shao H, Jiang H (2018). Intelligent fault diagnosis of rolling bearing using deep wavelet auto-encoder with extreme learning machine. Knowl. Based Syst..

[34] Mao W, Feng W (2019). A novel deep output kernel learning method for bearing fault structural diagnosis. Mech. Syst. Signal Process..

[35] Sobie C, Freitas C (2018). Simulation-driven machine learning: Bearing fault classification. Mech. Syst. Signal Process..

[36] Xue H, Li Z (2014). Intelligent diagnosis method for centrifugal pump system using vibration signal and support vector machine. Shock. Vib..

[37] Tang H, Liao Z (2021). A robust deep learning network for low-speed machinery fault diagnosis based on multi-kernel and RPCA. IEEE/ASME Trans. Mechatron..

[38] Shao S, McAleer S, Yan R, Baldi P (2018). Highly accurate machine fault diagnosis using deep transfer learning. IEEE Trans. Ind. Inform..

[39] Case Western Reserve University Bearing Data Center. Accessed 22 Dec 2019. https://csegroups.case.edu/bearingdatacenter/home.

[40] Yang S, Kong X, Wang Q, Li Z, Cheng H, Xu K (2022). Deep multiple auto-encoder with attention mechanism network: A dynamic domain adaptation method for rotary machine fault diagnosis under different working conditions. Knowl. Based Syst..

[41] Chen P, Zhao R, He T, Wei K, Yang Q (2022). Unsupervised domain adaptation of bearing fault diagnosis based on Join Sliced Wasserstein Distance. ISA Trans..

[42] Zhu Z, Peng G (2019). A convolutional neural network based on a capsule network with strong generalisation for bearing fault diagnosis. Neurocomputing.

[43] Han T, Li YF (2022). Out-of-distribution detection-assisted trustworthy machinery fault diagnosis approach with uncertainty-aware deep ensembles. Reliab. Eng. Syst. Saf..

[44] Tang H, Liao Z, Chen P, Zuo D, Yi S (2020). A novel convolutional neural network for low-speed structural fault diagnosis under different operating condition and its understanding via visualization. IEEE Trans. Instrum. Meas..

